# Comparison of TALEN scaffolds in *Xenopus tropicalis*

**DOI:** 10.1242/bio.20136676

**Published:** 2013-11-06

**Authors:** Keisuke Nakajima, Yoshio Yaoita

**Affiliations:** Division of Embryology and Genetics, Institute for Amphibian Biology, Graduate School of Science, Hiroshima University, Higashihiroshima 739-8526, Japan

**Keywords:** *Xenopus tropicalis*, TALENs, Genome editing, Targeted gene knockout

## Abstract

Transcription activator-like effector nucleases (TALENs) are facile and potent tools used to modify a gene of interest for targeted gene knockout. TALENs consist of an N-terminal domain, a DNA-binding domain, and a C-terminal domain, which are derived from a transcription activator-like effector, and the non-specific nuclease domain of FokI. Using *Xenopus tropicalis* (*X. tropicalis*), we compared the toxicities and somatic mutation activities of four TALEN architectures in a side-by-side manner: a basic TALEN, a scaffold with the same truncated N- and C-terminal domains as GoldyTALEN, a scaffold with the truncated N- and C-terminal domains and an obligate heterodimeric nuclease domain, and a scaffold with the truncated N- and C-terminal domains and an obligate heterodimeric *Sharkey* nuclease domain. The strongest phenotype and targeted somatic gene mutation were induced by the injection of TALEN mRNAs containing the truncated N- and C-terminal domains and an obligate heterodimeric nuclease domain. The obligate heterodimeric TALENs exhibited reduced toxicity compared to the homodimeric TALENs, and the homodimeric GoldyTALEN-type scaffold showed both a high activity of somatic gene modification and high toxicity. The *Sharkey* mutation in the heterodimeric nuclease domain reduced the TALEN-mediated somatic mutagenesis.

## Introduction

Gene knockout is an important method used to demonstrate the function of a specific gene. Targeted gene disruption by homologous recombination in embryonic stem (ES) cells is currently feasible only in mice (Chisaka and Capecchi, 1991) and rats (Tong et al., 2011) due to the difficulty of establishing ES cell lines from other species. In contrast, zinc-finger nucleases (ZFNs) consisting of a zinc-finger DNA-binding domain and the nuclease domain of the restriction enzyme FokI (Kim and Chandrasegaran, 1994), have been used to modify a gene of interest in many animal models. However, the design process of ZFNs is time-consuming and labor-intensive and available target sites are limited. Transcription activator-like effector nucleases (TALENs) are fusion proteins composed of a DNA-binding domain of a transcription activator-like effector and the nuclease domain of FokI, as in ZFNs (Christian et al., 2010; Li et al., 2011). TALENs are becoming powerful molecular tools because of their simple design (Boch et al., 2009; Moscou and Bogdanove, 2009) and rapid assembly (Cermak et al., 2011), and they have been successfully used for targeted mutagenesis in several species (Wood et al., 2011; Cade et al., 2012; Lei et al., 2012; Liu et al., 2012; Wang et al., 2013). Both ZFNs and TALENs work as dimers to cleave the target DNA sequence, which is then repaired by homologous recombination (Bibikova et al., 2001) or modified by non-homologous end-joining (Lukacsovich et al., 1994). The latter results in nucleotide insertion and/or deletion at the cleavage sites, frequently leading to a loss of gene function.

Multiple TALEN scaffolds have been reported in addition to the basic architecture, pTAL (Cermak et al., 2011). A pTAL-encoded TALEN protein contains a nuclear localization signal, an N-terminal domain (287 amino acids), a target DNA-binding domain composed of many tandem 34-amino-acid repeats, a C-terminal domain (231 amino acids), and the nuclease domain of FokI. An analysis of a series of truncation variants of pTAL revealed that a region encompassing approximately 150 amino acids upstream of the DNA-binding domain is essential for efficient binding to a target site, whereas the C-terminal domain is dispensable for the interaction with DNA (Miller et al., 2011; Mussolino et al., 2011; Zhang et al., 2011). GoldyTALEN was designed to contain a truncated N-terminal domain (158 amino acids) and C-terminal domain (63 amino acids) upstream and downstream, respectively, of the DNA binding domain, and has been shown to have greater somatic gene modification activity than the pTAL scaffold in zebrafish (Bedell et al., 2012). Furthermore, a lower toxicity and comparable or higher somatic mutation activity were reported in zebrafish using TALENs harboring an obligate heterodimeric FokI nuclease domain than using TALENs containing homodimeric FokI (Cade et al., 2012). We introduced the *Sharkey* mutation into this TALEN scaffold, as *Sharkey* is known to enhance the DNA cleavage activity of FokI in both homodimeric and heterodimeric ZFN architectures (Guo et al., 2010; Doyon et al., 2011). In the present study, we performed a side-by-side comparison of the somatic mutation activity and toxicity of these TALEN scaffolds using *Xenopus tropicalis* (*X. tropicalis*).

## Results

**Fig. 1.**
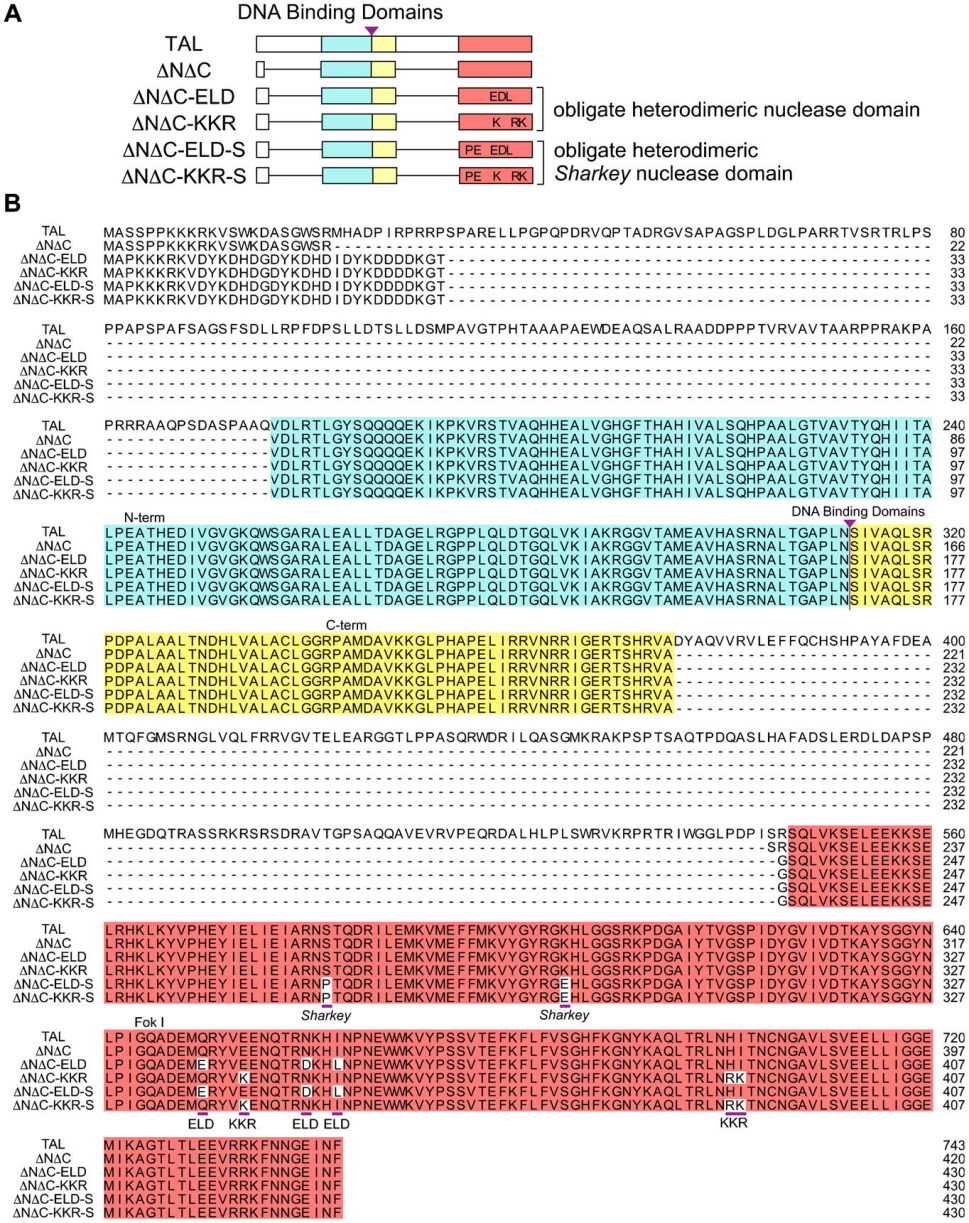
A comparison of TALEN scaffold structures. (A) A schematic representation of TAL, ΔNΔC, ΔNΔC-ELD/KKR and ΔNΔC-ELD-S/KKR-S. (B) A comparison of TALEN scaffold amino acid sequences. The amino acid exchanges in the nuclease domain of ΔNΔC-ELD/KKR and ΔNΔC-ELD-S/KKR-S are underlined. (A,B) The shared amino acids in the N-terminal domain and C-terminal domain of TALEN are indicated with blue and yellow boxes, respectively. The nuclease domain of FokI is indicated with a red box. A purple triangle denotes the insertion-site of the DNA binding domain.

**Fig. 2.**
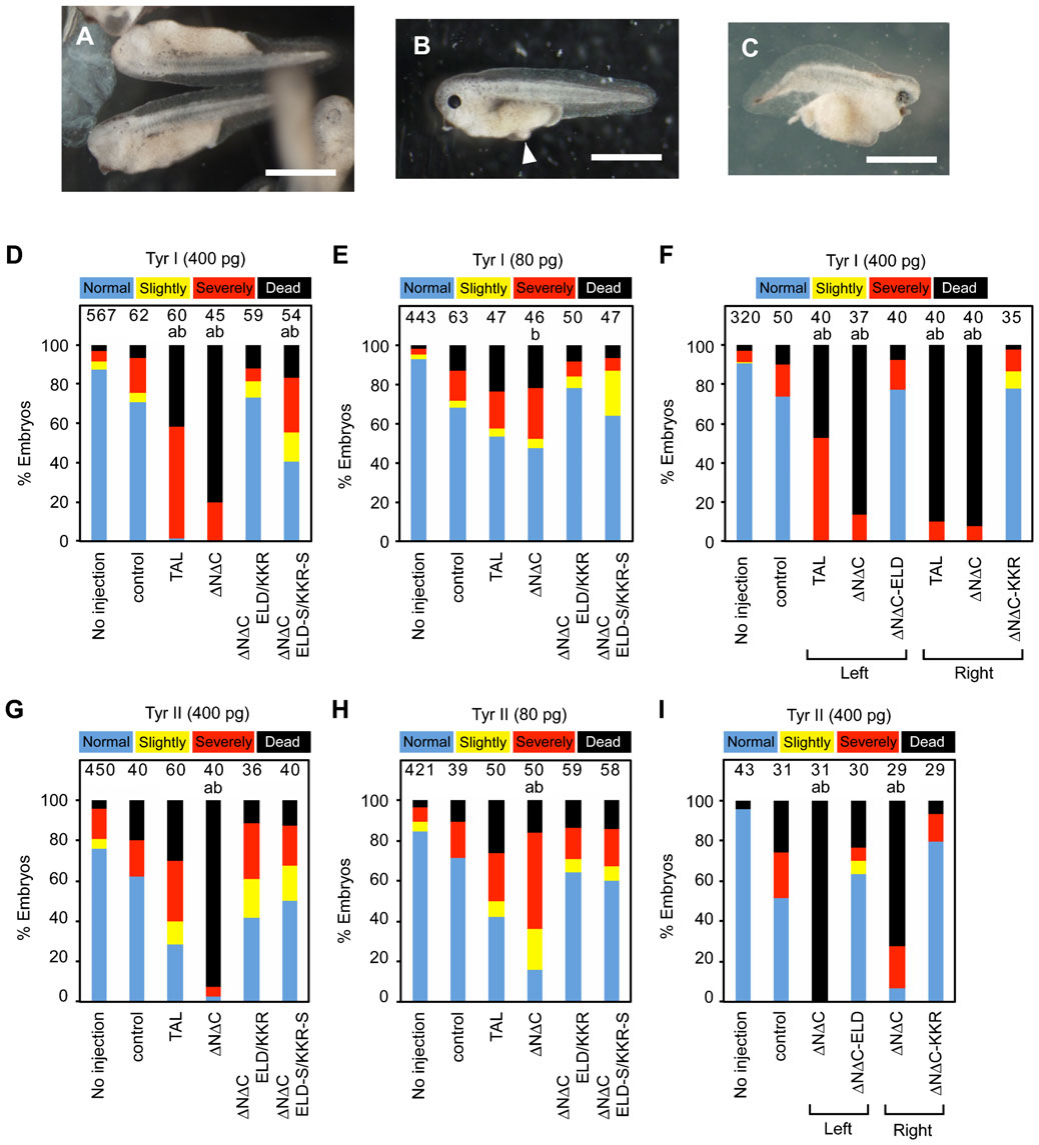
The toxicity of TALEN mRNAs in *X. tropicalis* embryos. (A) Morphologically normal embryos (Normal) with a loss of pigmentation in the retina after injection of ΔNΔC-ELD/KKR-Tyr I mRNAs. (B) A slightly deformed embryo (Slightly) that had not been injected with any mRNA. A small edema is indicated with a white arrowhead. (C) A severely deformed embryo (Severely) injected with ΔNΔC-ELD/KKR-Tyr I mRNAs. (D–I) Percentages of normal (blue), slightly deformed (yellow), severely deformed (red) and dead (black) embryos at NF-stage 35/36 (D,E,G,H) or NF-stage 24/25 (F,I). Embryos were injected with 400 pg (D,G), 80 pg (E,H) or 0 pg (control) of mRNAs encoding TAL, ΔNΔC, ΔNΔC-ELD/KKR or ΔNΔC-ELD-S/KKR-S TALEN for the Tyr I (D,E) or Tyr II (G,H) sites. (F,I) Embryos were injected with 400 pg of mRNA encoding TAL, ΔNΔC or ΔNΔC-ELD/KKR for the Tyr I left or right target site (F) and the Tyr II left or right target site (I). The number of embryos is indicated at the top of each column. The statistical significance compared to the control (a) or embryos injected with ΔNΔC-ELD/KKR mRNA (b) was assessed using a Steel-Dwass test. *P*<0.05. Scale bars: 1 mm.

We generated two pairs of DNA binding domains for targeted sites, Tyr I and Tyr II, in the first exon of the *tyrosinase* gene of *Xenopus tropicalis* (*X. tropicalis*) using the Golden Gate assembly method (Cermak et al., 2011). We selected this gene because tyrosinase is essential for melanin synthesis and the bi-allelic disruption of the *tyrosinase* gene results in an albino phenotype with white eyes and skin, which is easily discernible (Ishibashi et al., 2012; Nakajima et al., 2012). As shown in Fig. 1, these DNA binding domains were inserted into a basic TALEN vector (TAL) (Cermak et al., 2011), a TALEN scaffold with the same truncated N- and C-terminal domains as GoldyTALEN (ΔNΔC) (Bedell et al., 2012), a scaffold with the truncated N- and C-terminal domains and an obligate heterodimeric nuclease domain of FokI (ΔNΔC-ELD/KKR) (Doyon et al., 2011; Lei et al., 2012), and a scaffold with the truncated N- and C-terminal domains and an obligate heterodimeric *Sharkey* nuclease domain (ΔNΔC-ELD-S/KKR-S) (Guo et al., 2010). The nuclear localization signal is included in the N-terminus of all scaffolds, and a FLAG-tag is present at the N-terminus of the heterodimeric TALENs. The homodimerization of the nuclease domains is suppressed by the mutations Q337E, N347D, and I350L in ΔNΔC-ELD and by E341K, H388R, and I389K in ΔNΔC-KKR (Doyon et al., 2011). The *Sharkey* mutations S269P and K292E increase the nuclease activity of FokI within the context of both the homodimeric and heterodimeric ZFN architectures (Guo et al., 2010; Doyon et al., 2011). For a side-by-side analysis, TALEN-coding mRNAs were synthesized *in vitro* and injected into fertilized *X. tropicalis* eggs at the two-cell stage at a high (400 pg) or low (80 pg) dose (Young et al., 2011; Ishibashi et al., 2012). The morphology of the embryos injected with TALEN mRNAs was examined at the hatching stage (NF-stage 35/36), two days after injection. In all, 83% to 94% of the embryos survived after injection of the heterodimeric TALEN mRNAs, ΔNΔC-ELD/KKR or ΔNΔC-ELD-S/KKR-S, for the Tyr I and Tyr II target sequences, and more than half were normal or slightly deformed (Fig. 2). In contrast, 80% and 93% of the embryos died after injection of the high dose of ΔNΔC mRNAs for the Tyr I and Tyr II target sequences (ΔNΔC-Tyr I and -Tyr II mRNAs), respectively, and the surviving embryos exhibited a severely deformed morphology. Moreover, injection of the low dose of ΔNΔC mRNAs resulted in more dead and severely deformed embryos compared to the heterodimeric TALEN mRNAs. TAL was less toxic to the embryos than ΔNΔC, but it was more harmful than heterodimeric TALENs. These data show that obligate heterodimeric TALENs are less toxic than homodimeric TALENs, consistent with a previous report (Cade et al., 2012).

A pair of left and right TALENs binds to left and right target sites, respectively (Fig. 5). To examine the basis of homodimeric TALEN mRNA toxicity, 400 pg of left or right TALEN mRNA was injected into two-cell-stage embryos (Fig. 2F,I). The morphological examination of NF-stage 24/25 embryos revealed that injection with only one of a pair of homodimeric TALEN mRNAs was more harmful to the embryos compared to ΔNΔC-ELD or ΔNΔC-KKR mRNA, suggesting that homodimeric TALENs disrupt more off-target genes than heterodimeric TALENs.

**Fig. 3.**
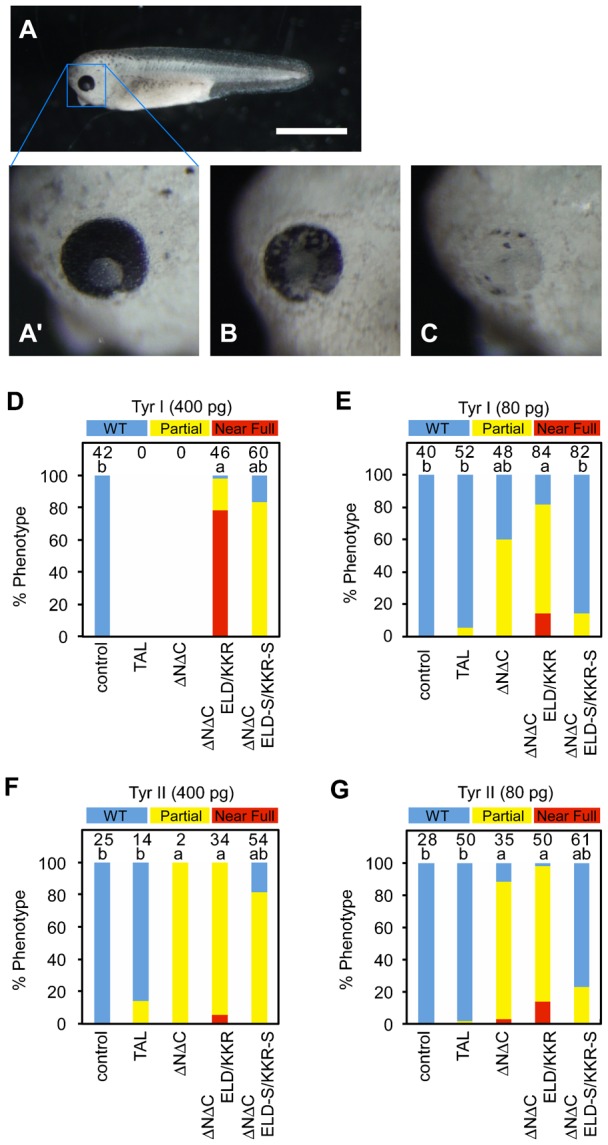
Phenotype of embryos injected with TALEN mRNAs. (A) A wild-type embryo at NF-stage 35/36. (A′) A higher magnification of A (WT). (B) An eye with less than 50% pigmentation loss in the retina (Partial). (C) An eye with more than 50% pigmentation loss in the retina (Near Full). (D–G) Percentages of wild-type eyes (blue), eyes with a partial loss (yellow), and eyes with a severe loss (red) of pigmentation in the retina of NF-stage 35/36 embryos injected with TAL, ΔNΔC, ΔNΔC-ELD/KKR or ΔNΔC-ELD-S/KKR-S mRNAs. The embryos were injected with 400 pg, 80 pg or 0 pg (control) of TALEN-Tyr I mRNAs (D,E) or TALEN-Tyr II mRNAs (F,G). The loss of retinal pigmentation was examined only in normal and slightly deformed embryos under the stereoscopic microscope. Almost all embryos injected with 400 pg of TAL-Tyr I or ΔNΔC-Tyr I mRNAs were dead or severely deformed and could not be analyzed (D). The number of eyes is indicated at the top of each column. The statistical significance compared to the control (a) or embryos injected with ΔNΔC-ELD/KKR mRNAs (b) was assessed using a Steel-Dwass test. *P*<0.05. Scale bar: 1 mm.

As black eyes are observable in wild-type embryos at the hatching stage, a loss of melanin in the retinal pigment epithelium was examined at NF-stage 35/36. Injecting the high dose of ΔNΔC-ELD/KKR-Tyr I mRNAs into the embryos resulted in the strongest albino phenotype, with most eyes showing near full albinism, suggesting that injection of the mRNAs caused the bi-allelic disruption of *tyrosinase* (Ishibashi et al., 2012; Nakajima et al., 2012; Suzuki et al., 2013) (Fig. 3). These embryos were reared to frogs, which also showed albinism (supplementary material Fig. S1). Injection of the low dose of ΔNΔC-ELD/KKR or ΔNΔC mRNAs also led to pigment loss in 60% to 98% of eyes. Conversely, the low dose of TAL-Tyr I and -Tyr II mRNAs induced pigment loss in only 5.8% and 2% of eyes, respectively, and even the high dose of TAL-Tyr II mRNAs elicited pigment loss in only 14% of eyes. This phenotype difference may be ascribed to the distinction in architecture between TAL and ΔNΔC, namely, the truncation of the N- and C-terminal domains flanking the DNA binding domain (Bedell et al., 2012) (Fig. 1). Unexpectedly, the *Sharkey* mutation in the heterodimeric nuclease domain did not enhance but rather appeared to impair the somatic mutagenesis of the *tyrosinase* gene by TALEN, particularly when the low dose of mRNAs was injected; this finding is in contrast to heterodimeric ZFN architectures (Doyon et al., 2011).

**Fig. 4.**
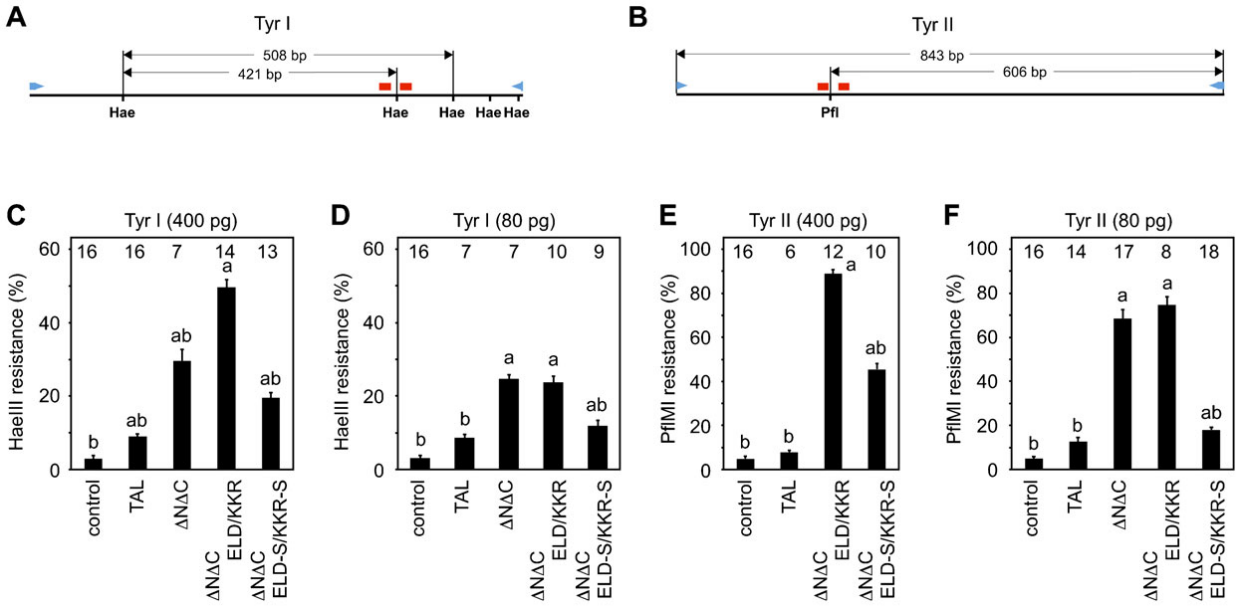
Disruption of restriction enzyme recognition sites between the left and right target sites in embryos injected with TALEN mRNAs. (A,B) Schematic drawing of the genomic PCR product containing Tyr I (A) or Tyr II (B) target sites. An HaeIII site (Hae) and a PflMI site (Pfl) are located in the spacer sequences of Tyr I and Tyr II, respectively. The primer sets and target sites are indicated as blue arrowheads and red bars, respectively. (C–F) Quantification of resistance to restriction enzyme digestion. Embryos were injected with 400 pg, 80 pg or 0 pg (control) of TALEN-Tyr I mRNAs (C,D) or TALEN-Tyr II mRNAs (E,F). Genomic DNA was separately prepared from randomly selected embryos at NF-stage 35/36 (Fig. 2; Fig. 3) and subjected to PCR using a specific primer set to amplify DNA fragments containing the target sites. The PCR products were digested with HaeIII (C,D) or PflMI (E,F) enzymes and separated on agarose gels. The injected TALEN scaffolds are shown at the bottom. The number of analyzed embryos is shown at the top of each column. Due to embryo death, genomic DNA was not extracted from embryos injected with 400 pg of ΔNΔC-Tyr II mRNAs (E). The statistical significance compared to the control (a) or embryos injected with ΔNΔC-ELD/KKR mRNAs (b) was assessed using a Tukey test. *P*<0.05. The data are expressed as the means ± s.e.m.

Somatic mutation by four types of TALEN scaffolds was quantified by measuring the loss of restriction enzyme recognition sites between target sites in the amplicons from a genomic DNA of the injected embryos. HaeIII and PflMI recognition sites are located in the spacers of the Tyr I and Tyr II sites, respectively (Fig. 4A,B). When these recognition sites are modified by error-prone non-homologous end-joining after cleavage by TALENs, the amplicons containing the target sequences become resistant to digestion by the restriction enzyme. Total cellular DNA was prepared from the injected embryos at NF-stage 35/36, and DNA fragments, including the TALEN target sites, were amplified by genomic PCR and subjected to restriction enzyme digestion and electrophoresis (Bedell et al., 2012). A quantitative analysis was accomplished by scanning the electrophoresis gels (supplementary material Fig. S2; Fig. 4C–F). The profiles of restriction enzyme digestion resistance are very similar to the patterns of the albino phenotype by TALEN mRNA injection (Fig. 3; Fig. 4). Resistance to restriction enzymes was significantly high in the amplicons derived from the embryos injected with ΔNΔC, ΔNΔC-ELD/KKR, or ΔNΔC-ELD-S/KKR-S mRNAs, and the resistance activity with ΔNΔC-ELD-S/KKR-S was significantly lower than that with ΔNΔC-ELD/KKR.

**Fig. 5.**
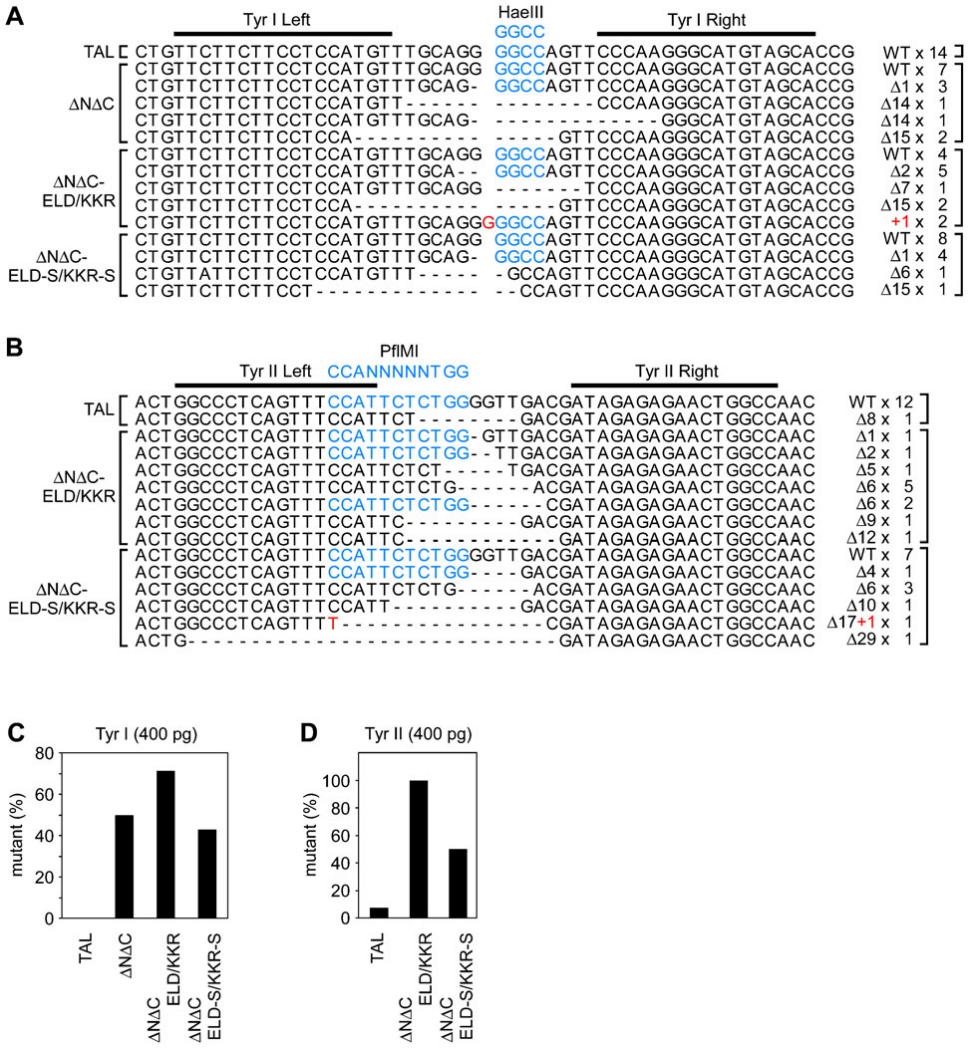
Target site sequences in TALEN-mRNA-injected embryos. Target DNA sequences were determined using pooled genomic DNAs purified from NF-stage 35/36 embryos that had been injected with 400 pg of TALEN-Tyr I (A) or –Tyr II (B) mRNAs. (A) Mutation of target site sequences by TALEN-Tyr I. Genomic DNA was extracted from each of 16, 9, 13 and 13 embryos injected with TAL-Tyr I, ΔNΔC-Tyr I, ΔNΔC-ELD/KKR-Tyr I and ΔNΔC-ELD-S/KKR-S-Tyr I mRNAs, respectively, and pooled. (B) Mutation of target site sequences by TALEN-Tyr II. Genomic DNA was extracted from each of 7, 12 and 9 embryos injected with TAL-Tyr II, ΔNΔC-ELD/KKR-Tyr II and ΔNΔC-ELD-S/KKR-S-Tyr II mRNAs, respectively, and pooled. Due to embryo death, genomic DNA was not purified from embryos injected with ΔNΔC-Tyr II mRNAs. The wild-type sequence is shown as WT. The black bars indicate the Tyr I (A) and Tyr II (B) target sites. Gaps resulting from deletion are denoted as dashes. Inserted nucleotides are indicated as red characters. The HaeIII (A) and PflMI (B) recognition sequences are indicated as blue characters. The mutation types and frequencies are indicated on the right. (C,D) Percentage of mutant target site sequences derived from embryos injected with TALEN-Tyr I (C) or TALEN-Tyr II (D) mRNAs.

Target site sequences were determined using a pool of genomic DNAs extracted at NF-stage 35/36 from randomly selected living embryos injected with the high dose of TALEN mRNAs (Fig. 5), though the genomic DNA from the ΔNΔC-Tyr II-mRNA-injected embryos was not available due to the high toxicity of ΔNΔC TALEN. The results support the presence of a TALEN activity that induces somatic gene modification based on the resistance to restriction enzyme digestion. The target site sequences were examined using genomic DNA from an embryo injected with ΔNΔC-ELD/KKR-Tyr I and -Tyr II mRNAs, and the mutation rates were 80% and 100%, respectively (supplementary material Fig. S3), consistent with those using pooled genomic DNAs. These data indicate that the restriction enzyme resistance assay has a tendency to underestimate mutation rates due to the conservation of restriction sites even after DNA repair.

## Discussion

In this study, we demonstrated in a side-by-side comparison that both the targeted somatic gene modification and the mutated phenotype are induced most efficiently in *X. tropicalis* embryos injected with TALEN mRNAs synthesized using a scaffold with the truncated N- and C-terminal domains and an obligated heterodimeric nuclease domain (Lei et al., 2012), ΔNΔC-ELD/KKR, with four types of architectures. In addition, injection of mRNAs encoding heterodimeric TALENs led to more healthy embryos compared to homodimeric TALENs. The ΔNΔC scaffold has a strong activity of somatic gene mutation similar to ΔNΔC-ELD/KKR, but it induces greater deformation and embryo death when the high dose of mRNAs is used, a result that is supported by the finding that several pairs of ΔNΔC showed high toxicity (data not shown). Although it may be possible to determine a dose of ΔNΔC mRNAs that exhibits a strong activity of somatic gene mutation with reduced toxicity, we believe that this dose is within a narrower range than that of the ΔNΔC-ELD/KKR mRNAs.

The injection of one of a pair of homodimeric TALEN mRNAs resulted in a high rate of severely deformed and dead embryos. We searched the *X. tropicalis* genome sequence Assembly v4.1 (Hellsten et al., 2010) for target sites for the Tyr I right and Tyr II left TALENs using the recognition sequences 5′-TRCTACATRCCCTTRRR-3′ and 5′-RRCCCTCARTTTCCAT-3′ (where R is A or G), respectively, because a TALEN DNA binding repeat that recognizes the nucleotide G also binds to the nucleotide A. We did not find any inverted repeats with 10 to 30 spacer nucleotides and three or fewer mismatched nucleotides. Instead, we identified 46 tandem repeats of a 36-nucleotide-long sequence in scaffold-170 (146100–147755), one repeat unit of which contains the recognition sequence (except the most 5′ nucleotide T) for the Tyr I right target site, and two tandem repeats with a spacer of 13 nucleotides in scaffold-26 (116679–116723), one repeat of which is the same sequence as the Tyr II left target site (except for one nucleotide mismatch). However, using a single-strand annealing assay (Nakajima et al., 2012), no activity was detected for the tandem repeats of scaffold-170 in cultured cells transfected with homodimeric TALEN-Tyr I right genes. Furthermore, no mutation near the Tyr II left target sequence in scaffold-26 was observed in 31 clones derived from the amplicon using genomic DNA from a ΔNΔC-Tyr II-left-mRNA-injected embryo (data not shown). The toxicity of homodimeric TALENs might be caused by non-specific genome-wide cleavage, as it is reported that many more double-strand breaks of genomic DNA are induced in cultured cells transfected with homodimeric ZFN genes than in those transfected with heterodimeric ZFN genes (Miller et al., 2007; Szczepek et al., 2007).

Genome editing is developing rapidly as a tool for targeted gene knockout in experimental animals, genome engineering of livestock and plants, and clinical gene therapy. Indeed, this rapid development is facilitated by the ease of design and assembly of TALENs using standard recombinant DNA techniques. As an illustration of this remarkable progress, several TALEN scaffolds are characterized herein, and more advanced TALEN scaffolds can be created with further research on each of these architectures.

## Materials and Methods

### Animals

The Ivory Coast line of *X. tropicalis* was provided by the National Bio-Resource Project of the Ministry of Education, Culture, Sports, Science and Technology, Japan. Fertilized eggs were obtained after injecting male and female *X. tropicalis* pairs with human chorionic gonadotropin (ASKA). The frogs were maintained at 24°C. The tadpoles were staged according to Nieuwkoop and Faber (Nieuwkoop and Faber, 1956). All animals were maintained and used in accordance with the guidelines established by Hiroshima University for the care and use of experimental animals.

### Construction of TALENs

The pTAL3 vector (Cermak et al., 2011) was digested with BglII and SacI to isolate a 2.7-kbp fragment, which was inserted into the multi-cloning site of pCMV-Script EX (dTRE) (Okada et al., 2012) to obtain a basic TALEN (TAL) under the control of the cytomegalovirus immediate early promoter. The constructed vector was named pCMV-TAL and used instead of pTAL3 or pTAL4. The N-terminus of TALEN was deleted from pCMV-TAL using inverted PCR with the primers Goldy-F1 and Goldy-R1 to create pCMV-TAL-dN (Table 1). A DNA fragment with the truncated N-terminal domain (158 amino acids) and C-terminal domain (63 amino acids) was amplified from this vector using PCR with the primers Goldy-F2 and Goldy-R2 and then digested with ApaI (Table 1). The ApaI-EcoRV fragment containing the full-length C-terminal domain of TALEN was replaced in pCMV-TAL-dN with the ApaI-digested PCR fragment including the truncated N- and C-terminal domains to produce a ΔNΔC TALEN (ΔNΔC). ΔNΔC and GoldyTALEN share the same truncated N-terminal domain (158 amino acids) and C-terminal domain (63 amino acids) (Bedell et al., 2012). TALEN scaffolds with the same truncated N- and C- terminal domains and obligate heterodimeric FokI (ELD/KKR) were a gift from Dr C. H. K. Cheng (Lei et al., 2012) and are referred to as ΔNΔC-ELD/KKR. The *Sharkey* mutation was introduced into the ΔNΔC-ELD/KKR scaffolds using inverted PCR with primers Sharkey-F and Sharkey-R (Table 1) to generate ΔNΔC-ELD-S/KKR-S (Guo et al., 2010).

**Table 1.**
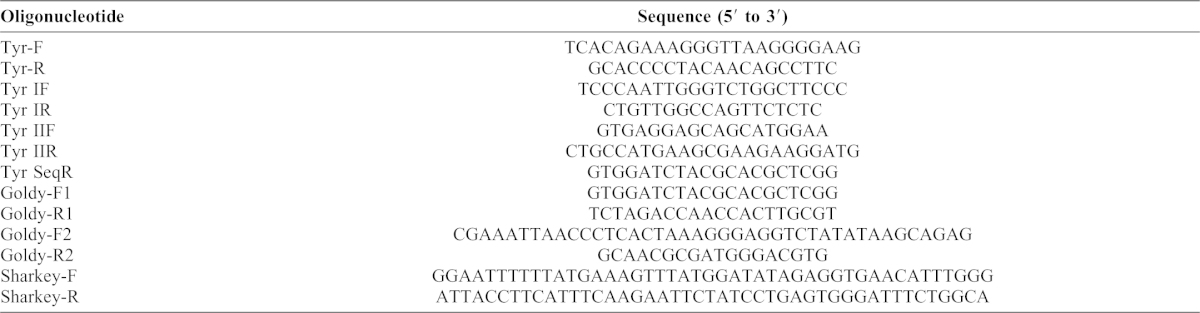
Oligonucleotide sequences used in the present study. The oligonucleotides were used for PCR and ΔNΔC and ΔNΔC-ELD-S/KKR-S construction.

Tyr I and Tyr II are the target sites in the first exon of the *tyrosinase* gene; Tyr I is described elsewhere (Ishibashi et al., 2012), and Tyr II corresponds to Tyr-B (Nakajima et al., 2013). The DNA binding domains were assembled as previously described (Cermak et al., 2011), with minor modifications as described elsewhere (Nakajima et al., 2013).

### RNA microinjection

TALEN mRNAs were transcribed *in vitro* using the mMESSAGE mMACHINE kits (Ambion). Each blastomere of two-cell-stage *X. tropicalis* embryos was injected with 2 nl of 50 or 10 ng/µl of each TALEN mRNA and 25 ng/µl of mCherry mRNA (Young et al., 2011; Ishibashi et al., 2012). The fluorescent product of the latter was used to identify the embryos that had been successfully injected and to confirm that the injected mRNA had been translated (Young et al., 2011). The embryos were reared at 22–24°C in 0.1× MMR with 0.1% BSA and 50 µg/ml gentamycin.

### Mutation analysis

Embryos were classified according to their morphology and the loss of pigmentation in the retinal pigment epithelium at NF-stage 35/36 (Nieuwkoop and Faber, 1956). Each individual embryo was homogenized in 90 µl of 50 mM NaOH and incubated for 10 min at 95°C. The homogenate was mixed with 10 µl of 1 M Tris-Cl (pH 8.0) and centrifuged at 1500 × g for 10 min at room temperature. The supernatant was treated with phenol and chloroform. DNA fragments of the target sites were amplified using EmeraldAmp MAX PCR Master Mix (TaKaRa) and Tyr-F and Tyr-R primers (Table 1), with 20 cycles of 95°C for 30 seconds, 58°C for 30 seconds, and 72°C for 2 min. The second PCR was performed using Tyr IF and Tyr IR for Tyr I or Tyr IIF and Tyr IIR for Tyr II (Table 1), with 20 cycles of 95°C for 30 seconds, 58°C for 30 seconds, and 72°C for 1 min. The PCR products were digested with HaeIII (TOYOBO) or PflMI (NEB) to examine the efficiency of targeted gene disruption in the injected embryos. The products were analyzed by gel electrophoresis, and the brightness of each band was measured; the molecular ratio was calculated as the quotient of the brightness divided by the nucleotide length. To confirm somatic mutations, the PCR products generated using TaKaRa EX Taq Hot Start Version (TaKaRa) with Tyr IIF and Tyr SeqR (Table 1) were subcloned into the pGEM-T Easy vector (Promega), and the nucleotide sequences were subsequently determined.

## Supplementary Material

Supplementary Material
