# Supplementary material for: Comparison of TALEN scaffolds in Xenopus tropicalis
Source: Biol Open. 2013 Nov 6;2(12):1364–70. doi: 10.1242/bio.20136676 (PMC3863421; doi:10.1242/bio.20136676)
Supplement: Supplementary Material [file supp_2_12_1364__index.html]

Comparison of TALEN scaffolds in Xenopus tropicalis — Comparison of TALEN scaffolds in Xenopus tropicalis — Supplementary Material 

# Comparison of TALEN scaffolds in *Xenopus tropicalis*

## bio.20136676 Supplementary Material

**Files in this Data Supplement:**

- Supplementary Material - Keisuke Nakajima and Yoshio Yaoita doi: 10.1242/bio.20136676
