# Supplementary material for: Comparison of TALEN scaffolds in Xenopus tropicalis
Source: Biol Open. 2013 Nov 6;2(12):1364–70. doi: 10.1242/bio.20136676 (PMC3863421; doi:10.1242/bio.20136676)
Supplement: Supplementary Material [file supp_bio.20136676_bio.20136676-s1.pdf]

Biology Open

Supplementary Material

Keisuke Nakajima and Yoshio Yaoita **doi: 10.1242/bio.20136676**

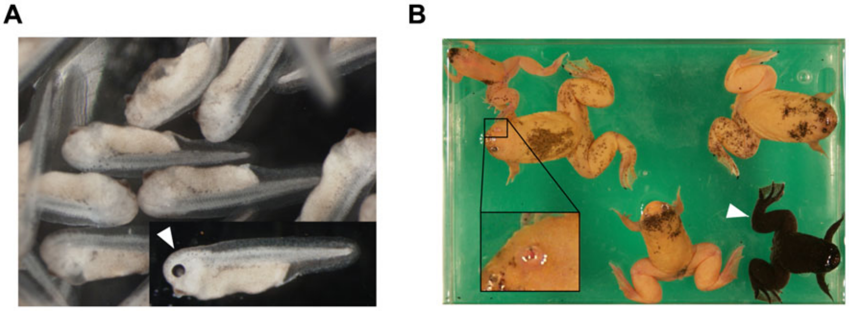

Fig. S1. Photographs of tadpoles and frogs that were raised from TALEN-mRNA-injected embryos. Embryos injected with ΔNΔC-ELD/KKR-Tyr I mRNAs were reared to albino tadpoles (A) and frogs (B). The white arrowheads indicate a wild-type tadpole (A) and a wild-type frog (B) that were bred from uninjected embryos. The inset shows the higher magnification of a right eye of an albino frog (B).

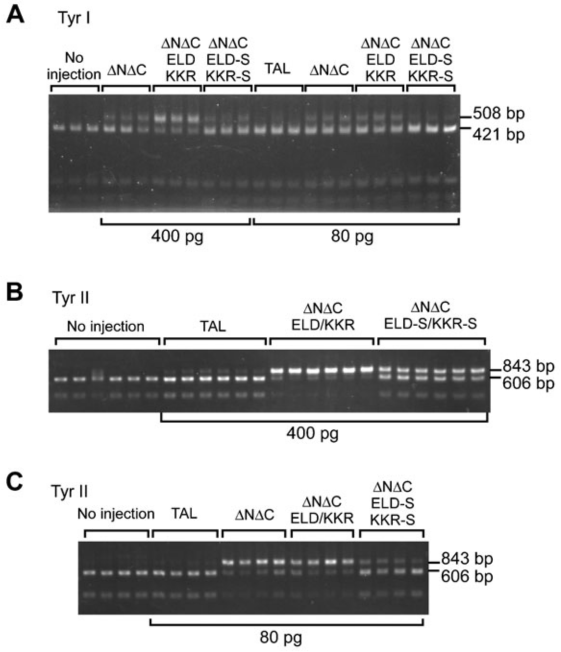

Fig. S2. Gel electrophoresis images of genomic PCR products from TALEN-mRNA-injected embryos after restriction enzyme digestion. Embryos were injected with 400 pg or 80 pg of TALEN-Tyr I mRNAs (A) and 400 pg (B) or 80 pg (C) of TALEN-Tyr II mRNAs. Genomic DNA from each embryo was prepared and subjected to PCR using a specific primer set to amplify a DNA fragment containing the target sites. The PCR products were digested using HaeIII (A) or PflMI (B,C). The injected TALEN scaffolds and doses of mRNA are shown at the top and bottom of the gel images, respectively. These images show the result from a portion of the embryos analyzed in Fig. 4.

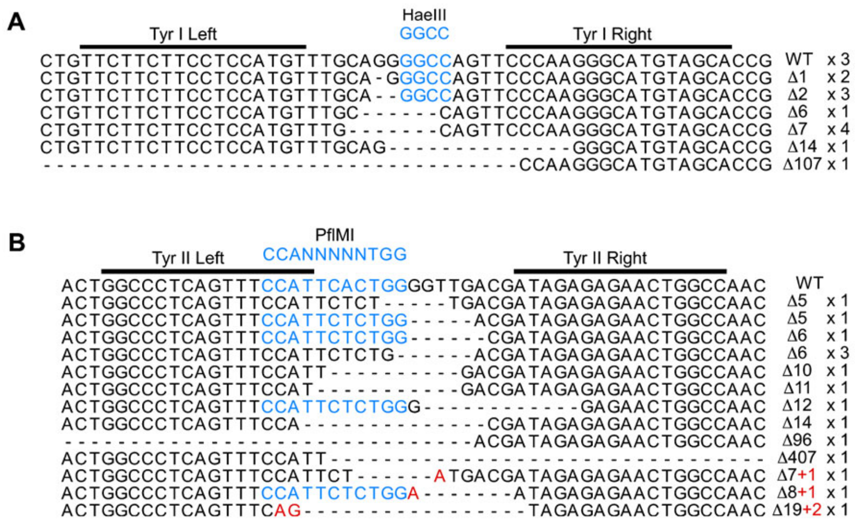

Fig. S3. Mutated target sequences in TALEN-mRNA-injected embryos. Target DNA sequences were determined using genomic DNA purified from an NF-stage 35/36 embryo that had been injected with 400 pg of ΔNΔC-ELD/KKR-Tyr I mRNAs (A) or -Tyr II mRNAs (B). The wild-type sequence is shown at the top. The black bars indicate the Tyr I (A) and Tyr II (B) target sites. Gaps resulting from deletion are denoted as dashes. Inserted nucleotides are indicated as red characters. The HaeIII (A) and PflMI (B) recognition sequences are indicated as blue characters. The mutation types and frequencies are indicated on the right.

Biology Open
